# In-house nucleic acid amplification tests for the detection of *Mycobacterium tuberculosis *in sputum specimens: meta-analysis and meta-regression

**DOI:** 10.1186/1471-2180-5-55

**Published:** 2005-10-03

**Authors:** Laura L Flores, Madhukar Pai, John M Colford, Lee W Riley

**Affiliations:** 1 Divisions of Infectious Diseases and Epidemiology, School of Public Health, University of California, Berkeley. CA 94720. USA; 2 Division of Molecular Biomedicine, CINVESTAV-IPN, Mexico DF, Mexico; 3 Division of Pulmonary & Critical Care Medicine, San Francisco General Hospital, San Francisco, CA 94110. USA

## Abstract

**Background:**

More than 200 studies related to nucleic acid amplification (NAA) tests to detect *Mycobacterium tuberculosis *directly from clinical specimens have appeared in the world literature since this technology was first introduced. NAA tests come as either commercial kits or as tests designed by the reporting investigators themselves (in-house tests). In-house tests vary widely in their accuracy, and factors that contribute to heterogeneity in test accuracy are not well characterized. Here, we used meta-analytical methods, including meta-regression, to identify factors related to study design and assay protocols that affect test accuracy in order to identify those factors associated with high estimates of accuracy.

**Results:**

By searching multiple databases and sources, we identified 2520 potentially relevant citations, and analyzed 84 separate studies from 65 publications that dealt with in-house NAA tests to detect *M. tuberculosis *in sputum samples. Sources of heterogeneity in test accuracy estimates were determined by subgroup and meta-regression analyses. Among 84 studies analyzed, the sensitivity and specificity estimates varied widely; sensitivity varied from 9.4% to 100%, and specificity estimates ranged from 5.6% to 100%. In the meta-regression analysis, the use of *IS6110 *as a target, and the use of nested PCR methods appeared to be significantly associated with higher diagnostic accuracy.

**Conclusion:**

Estimates of accuracy of in-house NAA tests for tuberculosis are highly heterogeneous. The use of *IS6110 *as an amplification target, and the use of nested PCR methods appeared to be associated with higher diagnostic accuracy. However, the substantial heterogeneity in both sensitivity and specificity of the in-house NAA tests rendered clinically useful estimates of test accuracy difficult. Future development of NAA-based tests to detect *M. tuberculosis *from sputum specimens should take into consideration these findings in improving accuracy of in-house NAA tests.

## Background

Accurate and early diagnosis of tuberculosis (TB) is a critical step in the management and control of TB. Because conventional tests for TB have several limitations, nucleic acid amplification (NAA) tests have emerged as promising alternatives. The polymerase chain reaction (PCR) is the best-known and most widely used NAA test. NAA tests are categorized as commercial (kit-based) or in-house ("home-brew"). In-house tests are those assays where the investigators design their own protocols. In-house tests are commonly used in developing countries where commercial kits may not be affordable. The accuracy of NAA tests for TB has been extensively studied since the early 1990s. Since hundreds of studies have evaluated the accuracy of NAA tests, it is now possible to evaluate their overall performance using meta-analysis methods and determine which study design or test-related characteristics are associated with higher diagnostic accuracy.

Several systematic reviews and meta-analyses have been published in the past few years on the accuracy of NAA tests for pulmonary and extra-pulmonary TB [1,5]. Because these meta-analyses and reviews have synthesized data from over 200 primary studies, and because their results are highly consistent with each other, they provide us with the best available evidence on the accuracy of NAA tests. The following are the main findings of the meta-analyses and reviews: most of the studies on NAA tests reported very high estimates of specificity, for both pulmonary and extra-pulmonary TB [1-5]. Sensitivity estimates, in contrast, have been much lower and highly variable (heterogeneous) [1-5]. Sensitivity estimates have been lower in paucibacillary forms of TB (smear-negative pulmonary and extra-pulmonary TB), and higher in smear-positive pulmonary TB [2,3]. Another striking result is the widespread lack of consistency in accuracy estimates across studies – studies have reported highly variable estimates of test accuracy [1-5]. For example, in our previous meta-analysis on NAA tests for tuberculous meningitis, sensitivity estimates varied between 0 – 100% [3]. In general, almost all the meta-analyses have demonstrated that the sensitivity and specificity of in-house PCR assays have been more variable and inconsistent than commercial tests [2,3,5].

Why do studies on in-house PCR assays produce such highly variable estimates of sensitivity and specificity? Is the variability due to differences in study design or to differences in assay characteristics and laboratory techniques? Are there specific study design features and assay characteristics that yield higher estimates of accuracy? Answers to these questions might help to identify features of NAA tests that maximize accuracy. However, these questions are difficult to address in individual studies. Meta-analytic methods, on the other hand, are well suited to explore the issue of why studies produce variable results. By synthesizing data from multiple studies and increasing the power of analyses, meta-analyses are able to employ techniques that help to identify sources of heterogeneity in study findings. In this meta-analysis, we reviewed 65 published studies on in-house NAA tests for pulmonary TB. The main objective of our meta-analysis was to determine factors associated with heterogeneity as well as higher accuracy estimates of accuracy in studies that evaluated in-house PCR for the diagnosis of pulmonary TB.

## Results

### Study selection

By searching multiple databases and sources we identified 2520 potentially relevant citations on NAA tests for tuberculosis. After screening titles and abstracts, 434 English and Spanish articles were selected for full-text review and 129 articles reported inclusion of sputum specimens tested by an in-house PCR assay. Sixty-one articles were then excluded mainly because data were not separately provided for sputum samples (sputum and other clinical specimens were analyzed together). Also, three articles were excluded because real time PCR was used and the number was insufficient to place them in a separate category. A total of 65 articles [10-74], were included in the final analysis. Four articles were in Spanish [19,41,46,53]. Thirteen articles reported evaluations of more than one NAA test against a common reference standard [11,13,14,20,28,36,47,54,60,62,68,69,72]. Each such test comparison was counted as a separate study. Thus, the total number of test comparisons (hereafter referred to as studies) was 84.

### Characteristics of included studies

The summary characteristics of the included studies are shown in Table 1. The average sample size of the included studies was 149 (range 14 to 727). Our data, as seen in Table 1, were affected by the poor quality of reporting in the primary studies. Fifty-five of 84 (65.5%) studies did not report blinded interpretation of NAA test independent of the reference standard, while only 29 of 84 (34.5%) reported single or double blinding of NAA test and the reference standard. Most of the studies, 60 of 84 (72%), were cross-sectional whereas 24 (28%) were case-control studies. The studies differed greatly in terms of laboratory characteristics. Fifty-four of 84 (65%) studies used *IS6110 *as amplification target by itself or in combination with other targets, and 30 studies (35%) used other targets (e.g. MPB64, 38 kDa). The studies were categorized as those using any chemical method for DNA extraction (including phenol-chloroform) and those in which any physical or mechanical extraction method was used. Sixty-eight of 84 (81%) studies reported a simple PCR protocol (including multiplex PCR), whereas 16 (19%) studies used a nested or seminested PCR protocol. Lastly, 49 of 84 (58%) studies used UV transillumination of an electrophoretic gel, and 35 (42%) used DNA hybridization probes to detect the amplification products.

### Overall diagnostic accuracy

**Figure 1:**
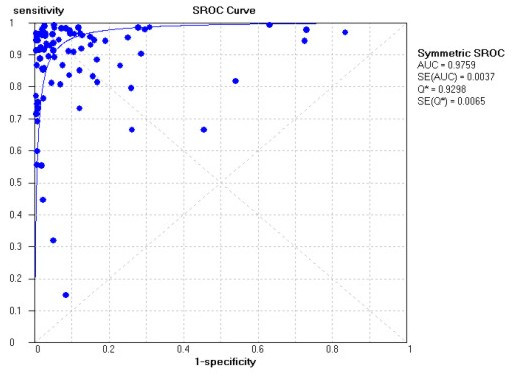
**Summary Receiver Operative Curve (SROC) for all studies**. Each solid circle represents each study in the meta-analysis. The regression line summarizes the overall diagnostic accuracy. Area under the curve (AUC) = 0.97.

When all 84 studies were evaluated together, the sensitivity estimates ranged from 9.4% to 100%, and specificity estimates ranged from 5.6% to 100%. Both measures were highly heterogeneous (P < 0.001 for test of heterogeneity). Figure 1 shows the overall accuracy of PCR in a summary receiver operating characteristic (SROC) plot. The symmetric curve shows a trade off between sensitivity and specificity. The area under the SROC curve was 97%, and summary DOR was 159.4, indicating high accuracy. However, the significant heterogeneity in sensitivity and specificity estimates precluded the determination of clinically useful summary measures.

### Exploration of heterogeneity

In order to identify factors associated with heterogeneity, we performed stratified (subgroup) analyses. Table 2 presents the study quality and assay factors assessed and their effect on the estimated summary Diagnostic Odds Ratio (DOR). As seen in Table 2, studies that did not report the use of blinding produced a DOR nearly 2.5 times higher than studies that reported blinded interpretation of index test and reference standard results. Studies with PCR tests that used a nested protocol had almost 2 times higher DOR than those using a regular PCR protocol. The use of *IS6110 *target in comparison with those using any other target for amplification showed a DOR 1.7 times higher than studies that used other targets. A similar result was obtained when studies that used UV transillumination of a gel were compared to those that used a probe for detection. Studies that used chemical reagents for DNA extraction produced DOR estimates that were about 1.12 times greater than studies that used physical methods, indicating that the use of chemicals (including phenol chloroform) does not significantly improve the test accuracy. Only five studies reported the analysis on smear negative samples. When stratified by smear status, no major difference was seen in the DOR. But this result may be due to the small number of studies reporting only smear negative samples for diagnosis.

As in our previous meta-analyses [2,3], none of the stratified analyses for DOR results fully explained the significant heterogeneity across studies in this review; the statistical tests for heterogeneity were significant even within the various strata (data not shown). Therefore, a meta-regression analysis was performed (Table 3) to simultaneously evaluate multiple covariates in the same analysis. The outcome of the regression analysis is reported as the Relative Diagnostic Odds Ratios (RDOR).

As shown in Table 3, studies that used *IS6110 *as target of amplification, and studies that used nested PCR methods produced RDOR that were significantly higher than those that used other amplification targets or PCR methods. We present the SROC curves for these subgroups in figures 3A and 3B for target and amplification technique, respectively, to show the trade off between sensitivity and specificity. Blinding, detection technique and smear status showed a slightly higher RDOR but they were not statistically significant in the final regression model. Chemical-based DNA extraction did not produce a significant RDOR, indicating that the use of any chemical reagent for DNA extraction did not substantially affect diagnostic accuracy. No difference was seen in DOR in those studies that used phenol-chloroform versus any other DNA extraction method (data not shown).

## Discussion

### Principal findings

Diagnostic methods and, therefore, control of tuberculosis would be greatly improved by the standardization and application of nucleic acid amplification tests. Our meta-analytical review of 84 in-house PCR-based studies to detect *M. tuberculosis *in sputum samples showed a summary receiver operator characteristic (SROC) of 97%, indicating an overall high accuracy of these tests. However, because of significant heterogeneity in sensitivity and specificity estimates, clinically meaningful estimates of accuracy could not be derived. Our analysis showed substantial variability in specificity and sensitivity estimates, and it is clear that in-house PCR tests produce highly inconsistent estimates of diagnostic accuracy. Heterogeneity was clearly evident in the results and could not been explained fully even after stratified analyses. Variability in study design, study quality, and differences in thresholds (cut-points) across studies might account for some of the observed heterogeneity. Nevertheless, the meta-regression analysis highlighted some variables that do appear to yield higher accuracy estimates. The use of *IS6110 *as the amplification target, and the use of a nested PCR protocol appear to enhance accuracy. It is therefore worth considering the inclusion of these elements in in-house PCR protocols. Our analyses also suggest that the methods used for DNA extraction and signal detection were not critical.

### Clinical implications

Because of the observed heterogeneity in sensitivity and specificity, it is difficult to determine clinically useful estimates of accuracy. On the other hand, our findings have some relevance for the clinical microbiology laboratory. Our results suggest that the use of *IS6110 *target sequence in the protocol, and the use of nested PCR methods appear to significantly increase the diagnostic accuracy of PCR. In our previous meta-analysis on NAA test for tuberculous pleuritis, we found that NAA tests that used *IS6110 *targets produced DOR estimates 2.5 times higher than tests that used other target sequences [48]. Lack of blinding has been found to be associated with higher accuracy in previous meta-analyses [49,53]. Nevertheless we did not find a significant effect in our meta-regression model. Our stratified analyses, however, did show that unblinded studies were associated with higher summary DOR than blinded studies. Previous empiric research [37] and our earlier meta-analyses [48,49] suggest that studies that use a case-control design tend to overestimate diagnostic accuracy. Surprisingly, study design had little impact on diagnostic accuracy in our current analyses. It is possible that laboratory factors (such as target sequence and amplification technique) had a much stronger impact on accuracy than study design features in our analyses.

### Limitations of the review

In our review, we found only five studies reporting the analysis of smear negative specimens. Therefore, we could not determine the effect of smear status on accuracy of PCR. Since clinical sputum specimens frequently include smear-negative samples, the conclusions made in this meta-analysis may not apply to studies that included a large number of smear-negative samples. The accuracy estimates for smear-negative specimens have mostly been derived from studies on commercial kits, which have shown high specificity but lower and variable sensitivity [53]. The US Food and Drug Administration (FDA) approved the use of specific commercial kits initially only for smear positive samples, and recently for smear negative specimens [75]. Our review also excluded more recent studies that used other protocols for the detection of amplified DNA, such as real time PCR. We found only three such studies, and hence they could not be subject to meta-analyses. In the future, such methods may prove to enhance NAA test accuracy.

### Implications for research

One test characteristic significantly associated with increased accuracy was the use of *IS6110 *as a target of amplification. *IS6110 *is present in the *M. tuberculosis *genome, usually as multiple copies, which helps to increase the sensitivity of a PCR test. Potential problem with using this target is that some strains from certain parts of the world lack this insertion sequence [76]. A possible solution may be to use more than one target. However, we found that multiplex PCR did not contribute to increase the diagnostic accuracy.

## Conclusion

In summary, this meta-analytical review of various protocols for PCR-based diagnosis of pulmonary TB identified a few factors associated with improved diagnostic accuracy, and others that did not make a substantial difference. Future development of NAA-based tests to detect *M. tuberculosis *from sputum specimens should take into consideration these test characteristics as a way to improve accuracy of in-house NAA tests to diagnose pulmonary TB.

## Methods

### Identification of studies

We searched the following databases: PUBMED (1985–2002), EMBASE (1988–2002), Web of Science (1990–2002), BIOSIS (1993–2002), Cochrane Library (2002; Issue 2), and LILACS (1990–2002). All searches were up to date as of August 2002. The PubMed search was repeated in March 2004, to cover recent literature. The *Journal of Clinical Microbiology*, a high-yield journal with respect to diagnostic studies was separately searched (1992–2003). The search terms used included "tuberculosis", "mycobacterium tuberculosis", "nucleic acid amplification techniques", "direct amplification test", "polymerase chain reaction", "ligase chain reaction", "molecular diagnostic techniques", "sensitivity and specificity", "accuracy", or "predictive value". Citations were searched from multiple databases as well as obtained from experts in the field and from manufacturers of commercial tests. Reference lists from primary and review articles were searched. English and Spanish articles were selected for final full-text review. Conference abstracts were excluded because they universally contained inadequate data to permit evaluation. This criterion had been used and reported in previous papers [3].

### Study eligibility

Our search strategy aimed to include all available studies on in-house NAA tests for direct detection of *M. tuberculosis *in sputum specimens. To be included in the meta-analysis, a study should have: 1) included at least one comparison of an in-house PCR with an appropriate reference standard (i.e. culture), for detection of *M. tuberculosis *complex; 2) provided sufficient information on sensitivity and specificity; 3) provided enough information to judge methodological quality of the study.

The following studies were excluded from the review: 1) case reports; 2) evaluation of NAA tests on animal specimens; 3) studies on use of PCR assays for typing of strains; 4) studies on use of PCR assays for determining drug resistance; 5) studies on use of PCR for detection of only non-tuberculosis mycobacteria and 6) studies using only commercial NAA kits.

### Data extraction

The final analyses included all available studies on in-house PCR tests for direct detection of *M. tuberculosis *in sputum specimens. Two reviewers (LLF and MP) determined study eligibility independently. After study selection, data were extracted from each included study using a standardized data extraction form.

The final set of English and Spanish articles was assessed by one reviewer (LLF) and a sample of these was assessed by a second reviewer (MP) to check accuracy in data extraction. For each study, the following quality criteria were scored as met or not: 1) independent comparison of NAA test against reference standard; 2) cross-sectional versus case-control study design; 3) blinded (single or double) interpretation of test and reference standard results. The test methodology criteria included: 1) species identification, 2) methodology used for DNA extraction 3) type of PCR performed (nested-seminested vs. regular, including multiplex), 4) amplification target (IS6110 vs. any other target) 5) method of detection of the final product (ultra-violet (UV) transillumination of an electrophoretic gel vs. use of labeled probes for DNA hybridization), 6) measures taken to avoid contamination and 7) inclusion of positive and negative controls in the assay. If no data on the above criteria were reported in the primary studies, we contacted the authors of the studies for such information. For the purposes of analyses, responses coded as "not reported" were grouped together with "not met".

Since discrepant analysis (where discordant results between test and culture results are resolved, *post-hoc*, using clinical data) may be a potential source of bias in NAA test assessments, we preferentially included unresolved data where available.

### Meta-analysis methods

We used standard methods recommended for meta-analyses of diagnostic test evaluations [6]. Data were analyzed using Stata (version 8) and Meta-Disc (version 1.1) software. Our analyses focused on the following measures of diagnostic accuracy: sensitivity (true positive rate [TPR]), specificity (1-false positive rate [FPR]), and diagnostic odds ratio (DOR). Sensitivity is the proportion positive test results among those with the target disease. Specificity is the proportion negative test results among those without the disease. The DOR is a single indicator of test accuracy [7] that combines the data from sensitivity and specificity into a single number. The DOR of a test is the ratio of the odds of positive test results in the diseased relative to the odds of positive test results in the non-diseased. The value of a DOR ranges from 0 to infinity, with higher values indicating better discriminatory test performance (higher accuracy). A DOR of 1.0 indicates that a test does not discriminate between patients with the disorder and those without it. DOR values lower than 1.0 suggest improper test interpretation (a greater proportion of negative test results in the group with disease). Mathematically, the DOR can be computed using any of the following equations [7]:

DOR = (TP/FN) / (FP/TN)

DOR = [sensitivity/(1 - specificity)] / [(1 - specificity)/specificity]

DOR = Positive likelihood ratio / Negative likelihood ratio

Each study in the meta-analysis contributed a pair of numbers: TPR and FPR. Since these measures are correlated and vary with the thresholds (cut points for determining test positives) employed in the individual studies, it is customary to analyze TPR and FPR proportions as pairs, and to also explore the effect of threshold on study results [6]. We summarized the joint distribution of sensitivity and specificity using the Summary Receiver Operating Characteristic (SROC) curve [8]. Unlike a traditional ROC plot that explores the effect of varying thresholds on sensitivity and specificity in a single study, each data point in the SROC space represents a separate study. The SROC curve is obtained by fitting a symmetric regression curve to pairs of TPR and FPR. The SROC curve and the area under it present an overall summary of test performance, and display the trade off between sensitivity and specificity. A shoulder-like ROC curve suggests that variability in thresholds employed could, in part, explain variability in study results. It also suggests a common, homogeneous underlying DOR that does not change with the diagnostic threshold. The area under the SROC curve is a global measure of overall test accuracy. An area under the curve of 100% indicates perfect discriminatory ability. Heterogeneity in meta-analysis refers to a high degree of variability in study results [9]. Such heterogeneity could be due to variability in thresholds (cut-points), disease spectrum, test methods, and study quality across studies [9]. In the presence of significant heterogeneity, pooled, summary estimates from meta-analyses are not meaningful. We investigated heterogeneity using a meta-regression analysis. The meta-regression analysis was an extension of the SROC model [8,9]. In this unweighted linear regression model, studies (and not patients or specimens) were the units of analysis. The DOR was the outcome (dependent) variable. The independent variables were the covariates that might be associated with the variability in the DOR. Based on our previous meta-analyses [1-3], the following covariates were specified *a priori *as potential sources of variability: study design, blinded interpretation of NAA test and reference standard, type of PCR test, target sequence amplified, use of probes to detect amplification products, and use of phenol-chloroform for DNA extraction. The results of the meta-regression model are expressed as relative diagnostic odds ratios (RDOR) [7,9]. An RDOR of 1.0 indicates that a particular covariate (e.g. blinded study design) does not affect the overall DOR. An RDOR >1.0 indicates that studies with a particular characteristic (e.g. those that employed a specific target sequence in the PCR) have a higher DOR than studies without this characteristic. For a RDOR <1.0, the reverse holds.

## Authors' contributions

LLF designed the study, carried out the study selection, data abstraction, analysis and drafted the manuscript. MP participated in study selection, checked data abstraction and analysis. JC participated in the design of the study and critically reviewed the manuscript. LWR provided input in study design and critically reviewed the manuscript.

## Figures and Tables

**Figure 2:**
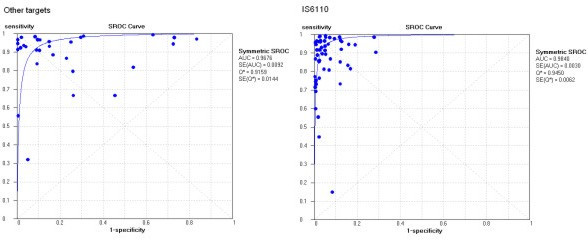
**Effect of Significant Test Characteristics on Summary ROC curves**: ROC curves for comparison among targets used in the in-house PCR assay (A) and different amplification techniques employed in the assays (B).

**Table 1:** Study characteristics and methodological quality of included studies

**Study characteristic**	**Frequency [N = 84 studies]**
**Study design**	
Cross sectional	60 (71%)
Case-control	24 (29%)
**Blinded assessment of NAA test and reference standard results**	
Double or single blinded	29 (34%)
Unblinded or Unknown	55 (66%)
**Target sequence for amplification**	
*IS6110*	54 (64%)
Other target sequences	30 (36%)
**Use of chemical-based DNA extraction method**	
Any chemical method	55 (66%)
Physical methods	29 (34%)
**Amplification technique**	
Nested or semi-nested PCR	16 (19%)
Regular or multi-plex PCR	68 (81%)
**Detection of amplified products**	
Probe-based method	49 (58%)
Gel-UV method	35 (42%)

**Table 2:** Stratified analysis: effect of study and test characteristics on summary diagnostic odds ratios

**Subgroup (Number of studies)**		**Summary DOR (95% CI)**
**Study design**	Case-control (24)	134.4 (65.2 – 213.1)
	Cross-sectional (60)	171.1 (106.7 – 274.2)
**Blinding**	Single or double blinded (29)	90.2 (46.2 – 176.2)
	Unblinded or NR (55)	221.2 (137.7 – 355.2)
**DNA extraction method**	Chemical (55)	153.7 (90.1 – 262.4)
	Physical (29)	171.7 (96.2 – 306.6)
**Amplification method**	PCR/multiplex (68)	139.3 (89.2 – 217.7)
	Nested PCR (16)	266.6 (140.8 – 504.6)
**Target sequence**	IS6110 (54)	236.1 (152.9 – 364.6)
	Other target (30)	77.4 (38.2 – 156.9)
**Detection method**	Probe (49)	130.6 (78.4 – 217.6)
	Gel-UV (35)	215.4 (115.1 – 403.2)
**Sputum smear status**	Pos/Both/NR (78)	161.5 (107.5 – 242.6)
	Negative (6)	128.6 (33.4 – 495.1)

**Table 3:** Meta-regression analysis to determine sources of heterogeneity

**Covariate**	**Coefficient**	**P-value**	**Relative diagnostic odds ratio (RDOR)**	**95% confidence interval**
**Intercept**	3.248	0.0052	----	----
**Threshold (S)**	- 0.072	0.4522	----	----
**Case control vs. cross-sectional design**	- 0.292	0.5219	0.75	(0.30 – 1.85)
**Blinded vs. unblinded studies**	0.441	0.2795	1.55	(0.69 – 3.48)
**Chemical vs. other DNA extraction methods**	- 0.209	0.5877	0.81	(0.38 – 1.74)
***IS6110 *vs. other target sequences**	1.055	0.0074	2.87	(1.34 – 6.16)
**Nested vs. regular amplification protocols**	1.196	0.0135	3.31	(1.29 – 8.49)
**Probe vs. gel-UV detection methods**	0.157	0.7222	1.17	(0.49 – 2.81)
**Sputum smear positive/both vs negative**	0.242	0.8146	1.27	(0.16 – 9.88)

Intercept: constant term in the model

S: indicator of threshold (logit TPR+logit FPR); TPR: true positive rate; FPR: false positive rate RDOR: relative diagnostic odds ratio (obtained by exponentiating the model coefficients)
